# Characterization and generation of human definitive multipotent hematopoietic stem/progenitor cells

**DOI:** 10.1038/s41421-020-00213-6

**Published:** 2020-12-01

**Authors:** Yanling Zhu, Tianyu Wang, Jiaming Gu, Ke Huang, Tian Zhang, Zhishuai Zhang, He Liu, Jun Tang, Yuchan Mai, Yanqi Zhang, Yuhang Li, Yashu Feng, Baoqiang Kang, Jinbing Li, Yongli Shan, Qianyu Chen, Jian Zhang, Bing Long, Junwei Wang, Minghui Gao, Di Zhang, Min Zhou, Xiaofen Zhong, Jiekai Chen, Duanqing Pei, Jinfu Nie, Bing Liu, Guangjin Pan

**Affiliations:** 1 CAS Key Laboratory of Regenerative Biology, Joint School of Life Sciences, Guangzhou Institutes of Biomedicine and Health, Chinese Academy of Sciences, Guangzhou Medical University, Guangzhou, Guangdong 510530 China; 2 Guangdong Provincial Key Laboratory of Stem Cell and Regenerative Medicine, South China Institute for Stem Cell Biology and Regenerative Medicine, Guangzhou Institutes of Biomedicine and Health, Chinese Academy of Sciences, Guangzhou, Guangdong 510530 China; 3 University of Chinese Academy of Sciences, Beijing, 100049 China; 4 Department of Hematology, The Third Affiliated Hospital, Sun Yat-sen University, Guangzhou, Guangdong 510630 China; 5 State Key Laboratory of Experimental Hematology, Fifth Medical Center of Chinese PLA General Hospital, Beijing, 100071 China; 6 Hefei Institute of Stem Cell and Regenerative Medicine, Guangzhou Institutes of Biomedicine and Health, Chinese Academy of Sciences, Guangzhou, Guangdong 510530 China; 7 Institute for Stem Cell and Regeneration, Chinese Academy of Sciences, Beijing, 100101 China

**Keywords:** Cell biology, Haematopoietic stem cells, Developmental biology

## Abstract

Definitive hematopoiesis generates hematopoietic stem/progenitor cells (HSPCs) that give rise to all mature blood and immune cells, but remains poorly defined in human. Here, we resolve human hematopoietic populations at the earliest hematopoiesis stage by single-cell RNA-seq. We characterize the distinct molecular profiling between early primitive and definitive hematopoiesis in both human embryonic stem cell (hESC) differentiation and early embryonic development. We identify CD44 to specifically discriminate definitive hematopoiesis and generate definitive HSPCs from hESCs. The multipotency of hESCs-derived HSPCs for various blood and immune cells is validated by single-cell clonal assay. Strikingly, these hESCs-derived HSPCs give rise to blood and lymphoid lineages in vivo. Lastly, we characterize gene-expression dynamics in definitive and primitive hematopoiesis and reveal an unreported role of ROCK-inhibition in enhancing human definitive hematopoiesis. Our study provides a prospect for understanding human early hematopoiesis and a firm basis for generating blood and immune cells for clinical purposes.

## Introduction

In embryogenesis, definitive hematopoiesis generates hematopoietic stem cells and multipotent progenitors (HSC/MPPs) that further give rise to all mature blood and immune cells. Classically, the multi-potent HSPCs undergo a stepwise differentiation to the oligo-, bi- and uni-potent progenitors of different types of mature blood cells, such as granulocyte-monocyte progenitors (GMPs), megakaryocyte-erythroid progenitors (MEPs), and lymphoid progenitors (LPs)^1–7^. However, many studies show that the uni-potent progenitors derive directly from HSPCs without going through oligo-potent progenitors, and thus the classical model of human hematopoiesis is revised^1,8–11^. Nonetheless, the multipotent HSC/MPPs that conceptually initiate hematopoiesis are very rare in adult human blood system^9,12,13^. Human HSCs and MPPs are phenotypically known to locate within the Lin^−^CD34^+^CD38^−^ compartment and both belong to a continuum of undifferentiated hematopoietic stem/progenitor cells (HSPCs)^6,8,9,12,13^.

During embryonic development, the multipotent HSPCs are originated in early definitive hematopoiesis through endothelial-to-hematopoietic transition (EHT)^14–18^. In the mouse model, the first wave hematopoiesis starts in yolk sac to generate primitive progenitors (mainly myelo-erythroid) and then in aorta-gonad-mesonephros (AGM) region to generate definitive HSPCs^14–19^. These two waves of hematopoiesis generate functionally distinct progenitors, and the immune cells such as NK, lymphoid cells are mainly generated from the second wave of definitive hematopoiesis. To this end, human primitive and definitive hematopoiesis have not been well-characterized in early embryonic development. Human pluripotent stem cells (PSCs) such as embryonic stem cells (ESCs) and induced pluripotent stem cells (iPSCs) are useful models for investigation of human hematopoietic development in vitro^20–23^. In hPSC differentiation, hemogenic endothelial cells (HECs) express CD34 and CD31 and then show upregulated CD43 on the commitment of hematopoietic fate^22,24–26^. CD43 has served as a marker to indicate hPSC-derived hematopoietic progenitors in many studies^23,24,27,28^. However, even though these CD43^+^ hPSC-derived HPCs could form various blood CFUs (colony-forming units), they have very limited definitive potency to give rise to immune cells^23,25^. Characterization of human primitive and definitive hematopoiesis at the earliest stage remains unaddressed, thus largely impeding the translational progress of human stem cell differentiation.

Here in this study, we resolve human early hematopoietic populations by single-cell RNA-seq and reveal distinct transcriptional profiling in early definitive and primitive hematopoiesis both in human embryonic stem cell (hESC) differentiation and early embryonic development. We identify CD44 to specifically discriminate the early definitive and primitive hematopoiesis. In addition, we generate definitive HSPCs from hESCs that give rise to various blood and immune lineages, including myeloid, erythroid/megakaryocyte cells, NK, T lymphocytes etc. and strikingly differentiate to various blood and lymphoid cells in vivo. Our studies provide a valuable model to understand human early hematopoietic development and generate various blood and immune cells for clinical purposes.

## Results

### Single-cell transcriptional profiling of hematopoietic populations in hESC differentiation

**Fig. 1:**
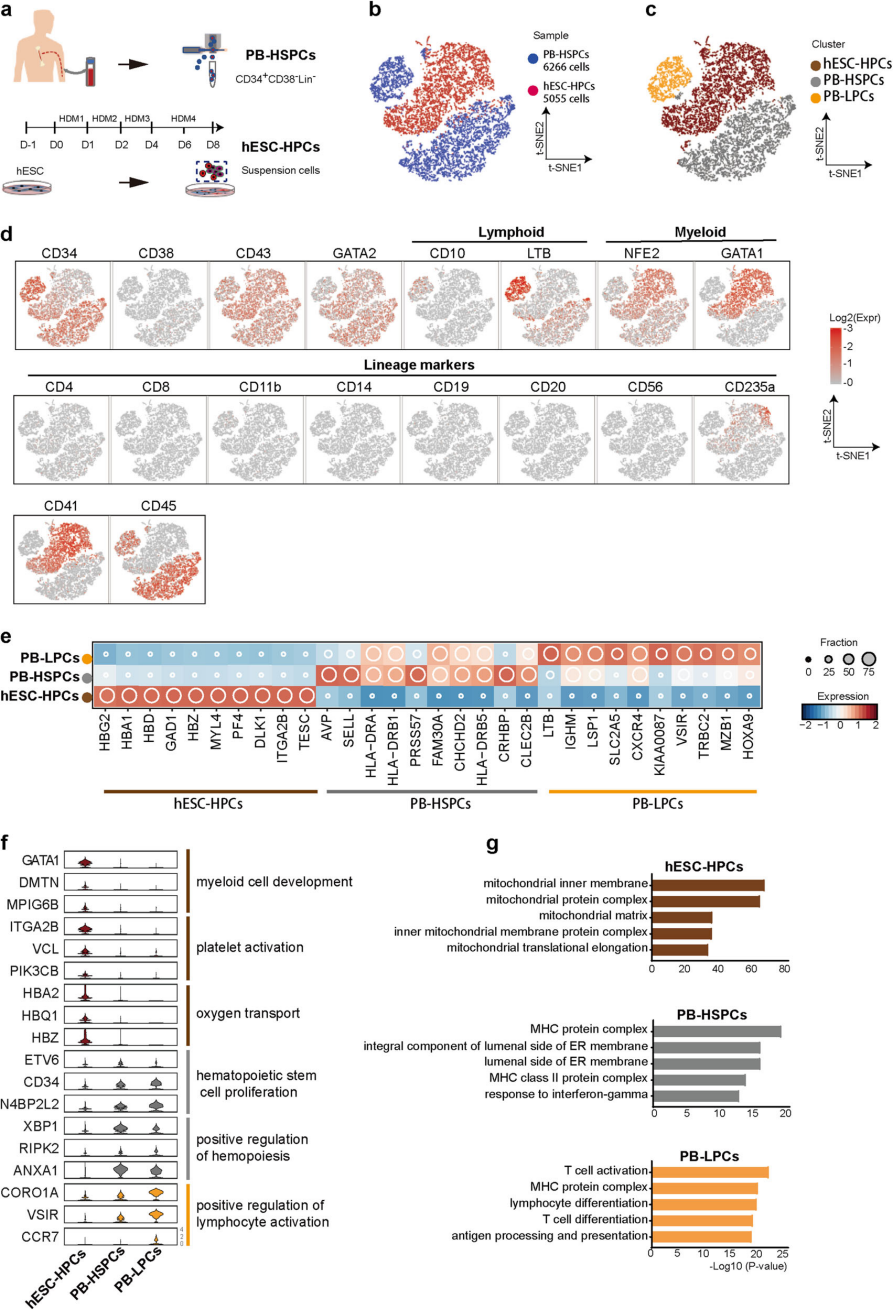
Single-cell RNA sequencing of hPSCs-derived HPCs and adult peripheral blood HSPCs. **a** Scheme of the experiment design. Fresh Lin^−^CD34^+^CD38^−^ HSPCs mobilized in human peripheral blood (PB-HSPCs) were sorted by FACS. Human ESCs were differentiated into blood lineages in a monolayer, defined condition. The floating blood cells at differentiation day 8 were sorted by CD43. The sorted cells were analyzed by 10× genomics for single-cell RNA sequencing (scRNA-seq). **b** *t*-SNE projection of PB-HSPC and hESC-HPC sample cells assigned based on samples. Each dot represents one cell and colors represent cell samples. **c** *t*-SNE projection of hESC-HPC, PB-HSPC, and PB-LPC cluster cells assigned based on clusters. Each dot represents one cell and cells are colored according to their assigned clusters. **d** Expressions of known hematopoietic and blood lineage marker genes at single-cell resolution. Color displays expression level (TPM, Log-scaled). **e** Expression profiles of top highly expressed genes in hESC-HPC, PB-HSPC and PB-LPC clusters. Color displays the scaled expression level (TPM, z-normalized) and diameter denotes fractional expression. **f** Violin plots show expression levels (TPM, Log-scaled) of selected hematopoietic and blood lineage marker genes with GO terms enriched in hESC-HPC, PB-HSPC and PB-LPC clusters. Colors represent clusters. **g** Top Gene Ontology (GO) terms enriched in hESC-HPC, PB-HSPC, and PB-LPC clusters. Colors represent clusters. The length of bar represents *P* value.

Based on reports from multiple groups^20,29,30^, we previously developed a stepwise strategy for hPSC blood differentiation in a totally defined, monolayer condition, which allows a robust EHT to generate CD43^+^ hematopoietic progenitors (hESCs-HPCs) that could form various blood CFUs^28^. However, the multi-potency of CD43^+^ hESCs-HPCs in hematopoiesis both in vitro and in vivo remains questionable. To address this issue, we generated single-cell transcriptional profiling on FACS-sorted CD43^+^ hESCs-HPCs differentiated from H1 hESCs at day 8 based on the previous protocol^28^ (Fig. 1a). As a comparison, we also sequenced single cells of FACS-sorted Lin^−^CD34^+^CD38^−^ HSPCs mobilized in human peripheral blood (PB-HSPCs) (Fig. 1a). *t*-SNE visualization on single-cell transcription profiling displayed distinct clusters between PB-HSPCs and hESCs-HPCs (Fig. 1b, c). Consistent with previous findings^9,13^, most Lin^−^CD34^+^CD38^−^ PB-HSPCs formed continuous rather than discrete pattern in transcriptional profiling (Fig. 1b, c). A small number of PB-HSPCs formed a separate cluster and expressed early lymphoid lineage markers such as CD10 and LTB^5^ (Fig. 1c, d), indicating they are lymphoid-primed HSPCs localized in Lin^−^CD34^+^CD38^−^ compartment (PB-LPCs)^6^. The FACS-sorted CD43^+^ hESCs-HPCs formed an indiscrete cluster separated from both PB-HSPCs and PB-LPCs (Fig. 1b, c). Consistent with the FACS sorting, hESCs-HPCs displayed a uniform CD43 expression and were negative for most known lineage markers of different mature blood cells (Fig. 1d). Surprisingly, most of these CD43^+^ hESCs-HPCs highly expressed *GATA1*, a known transcription factor for early erythroid lineage (Er)^31^, and *NFE2*, a myeloid (My) marker gene^32^ (Fig. 1d), indicating they might be Er/My-biased progenitors rather than the multipotent ones. Indeed, genes for myeloid development such as *MYL4*, *DMTN*, and *MPIG6B* and platelet and erythroid development genes like *ITGA2B*, *VCL*, *GATA1*, *HBG*, and *HBD* were highly enriched in hESC-HPC cluster (Fig. 1e, g). In contrast, known genes for definitive HSCs and/or lymphoid were highly enriched in PB-HSPC and PB-LPC clusters, respectively, such as *ETV6*, *CD34*, *HLADR*, *LTB*, *HOXA9*, and *CCR7* (Fig. 1e, g). The top gene ontology (GO) terms enriched in hESC-HPC cluster were related to mitochondrial regulation, reflecting this cluster is related to erythroid lineage (Fig. 1g). Consistently, the function of lymphoid regulation was significantly enriched in PB-LPCs (Fig. 1g). Together, we reveal that the marker-pure CD43^+^ hESCs-derived HPCs are mainly Er/My-biased progenitors rather than definitive ones based on gene-expression profiling, which has not been recognized before.

### hESCs-derived HPCs contain cells with different hematopoietic states

**Fig. 2:**
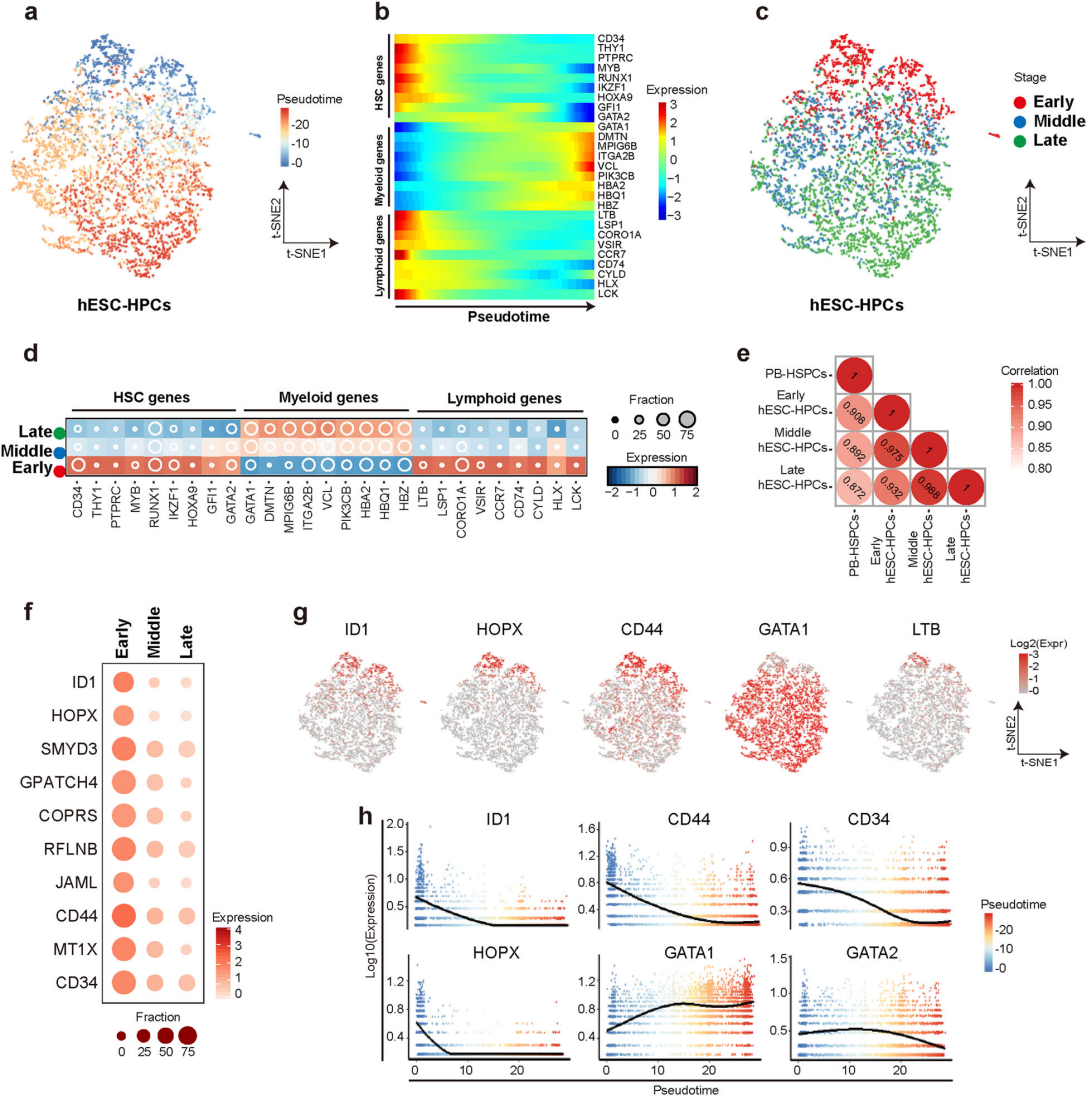
hPSCs-derived HPCs contain cells with different haematopoiesis states. **a** *t*-SNE projection of pseudotime order of hESC-HPC sample cells. Each dot represents one cell and cells are colored based on their putative pseudotime values. **b** Expression profiles of selected hematopoietic, myeloid and lymphoid marker genes along with the pseudotime progression of hESC-HPC sample. Color displays the scaled expression level (TPM, z-normalized). **c** *t*-SNE projection of hESC-HPC sample cells assigned to different stages based on pseudotime progression. Each dot represents one cell and cells are colored according to their assigned stages. **d** Expression profiles of selected hematopoietic, myeloid, and lymphoid marker genes in hESC-HPC sample at different stages of pseudotime progression. Color displays the scaled expression level (TPM, z-normalized) and diameter denotes fractional expression. **e** Pearson correlation between PB-HSPC sample cells and hESC-HPC sample cells at different pseudotime stages. Color represents coefficient of correlation computed based on bulk profiles merged from single cells. **f** Expression profiles of top highly expressed genes in hESC-HPC sample cells at different pseudotime stages. Color displays expression level (TPM, Log-scaled) and diameter denote fractional expression. **g** Expression profiles of selected blood marker genes in hESC-HPC sample. Each dot represents one cell and color displays expression level (TPM, Log-scaled). **h** Expression profiles of selected blood marker genes in hESC-HPC sample along the pseudotime. Each dot represents one cell and color represents pseudotime value. Line shows mean expression level (TPM, Log-scaled).

To further characterize the CD43^+^ hESCs-derived HPCs in detail, we applied pseudotime analysis to order all the hESC-HPCs based on their transcriptional profiling (Fig. 2a). Known genes that encode markers or regulators for human HSPCs such as *CD34*, *CD90*, *MYB*, *RUNX1*, *GFI1*, *IKZF1*, *HOXA9*, and *GATA2* were downregulated along the pseudotime, whereas genes that encode the Er/My regulators such as *GATA1*, *DMTN*, *VCL*, and *HBA* were significantly upregulated (Fig. 2b). Strikingly, genes with the function of lymphoid regulation such as *LTB*, *LSP1*, *CCR7*, were also downregulated along the pseudotime (Fig. 2b), indicating loss of definitive potential along pseudotime. We then divided hESC-HPCs into different clusters based on pseudotime progression (Fig. 2c). Consistently, genes that encode markers or regulators of HSCs such as *CD34*, *CD90*, *MYB*, *RUNX1*, *GFI1*, *IKZF1*, *HOXA9*, and *GATA2* were highly expressed in the early stage of cells but significantly downregulated in the cells at the middle and late stage in pseudotime progression (Fig. 2d). In contrast, the Er/My genes were expressed at lower levels in early stage cells, but their expression significantly increased in middle and late-stage cells (Fig. 2d). Again, lymphoid genes were highly expressed in the cells at an early stage but their high expressions were lost at later stages in pseudotime progression (Fig. 2d). These data indicate that cells at the early stage of pseudotime progression are less-primed definitive hematopoietic progenitors. Indeed, pearson co-efficiency analysis clearly showed a much closer relationship between the early stage hESC-HPCs and PB-HSPCs, compared with the cells at the middle and late stages (Fig. 2e). Top dynamic genes that were downregulated at later stage cells include those encode many known HSPCs or stem cell regulators such as *ID1*, *CD34*, *GATA2*, *HOPX*, and also those encode other new factors (Fig. 2f‒h). Notably, *GATA1*, the known Er regulator gene displayed a mutually exclusive pattern with *LTB*, the lymphoid gene, and most of the other HSPC-related genes along pseudotime progression (Fig. 2g‒h), again demonstrating the FACS-sorted CD43^+^ marker-pure hESCs-HPCs are heterogeneous and contain cells with different hematopoietic states.

### Identification of definitive multi-potent HSPCs in hESC differentiation

**Fig. 3:**
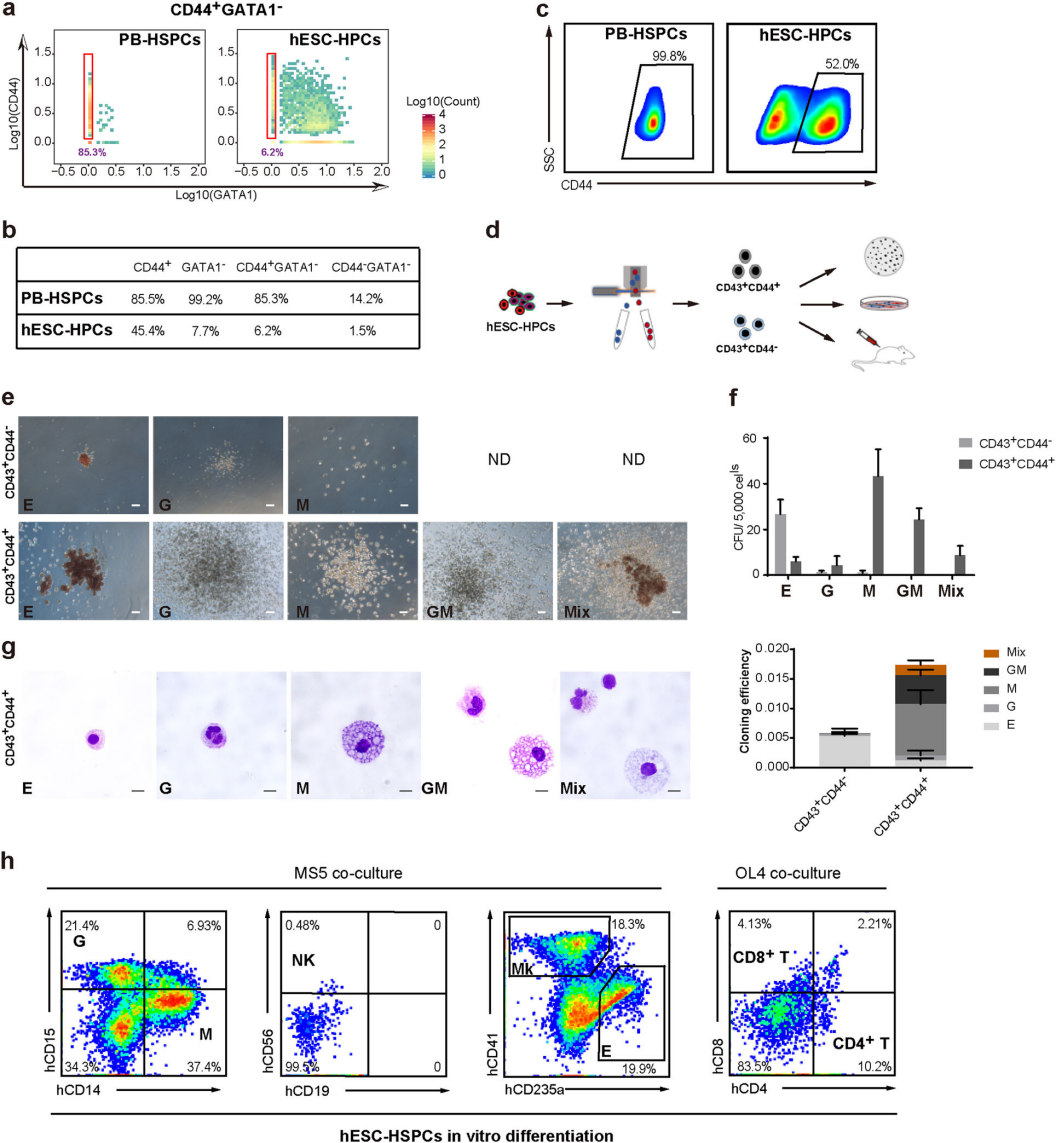
Identification of definitive multi-potent HSPCs in hESC differentiation. **a** Digital cytometry analysis on hESC-HPC and PB-HSPC sample cells based on CD44 and GATA1 expressions. Gate shows the fraction of CD44^+^GATA1^−^ cells in PB-HSPC and hESC-HPC samples. Colors represent density of cells. **b** Frequencies of cells of hESC-HPCs or PB-HSPCs based on expressions of CD44 and/or GATA1. **c** FACS analysis of CD44 expression in PB-HSPCs and hESC-HPCs. **d** Experimental strategy for functional characterization of hESC-HPCs. **e** Representative pictures of CFUs formed by hESC-HPCs sorted by CD43^+^CD44^+^ or CD43^+^CD44^−^. Scale bar, 100 μm; ND, not detected; E, erythroid; G, granulocytes; M, macrophages; GM, granulocyte and monocyte-macrophage; Mix, mixed erythro-myeloid. **f** Quantitative analysis of various blood CFUs formed by the CD43^+^CD44^+^ or CD43^+^CD44^−^ hESC-HPCs sorted at differentiation day 8. **g** May–Grunwald–Giemsa staining of different blood cells isolated from CFUs derived from CD43^+^CD44^+^ hESC-HPCs. Scale bar, 10 μm. **h**: Flow cytometry analysis on lineage output (indicated in quadrants) of CD44-sorted hESC-HPCs at differentiation day 8 co-cultured with MS5 or OP9-DL4 (OL4). The human hematopoietic cells were gated 2 weeks (MS5) or 4 weeks (OL4) after differentiation and analyzed for known lineage markers as indicated. G, granulocytes; M, macrophages; Mk, megakaryocytes; E, erythroid; NK, nature killer cells; T, T cells.

Since the cell surface marker *CD44* and *GATA1* are almost mutually exclusive in their expression and display opposite patterns during pseudotime progression (Fig. 2g), we reasoned that CD44 might specifically label the low-primed definitive HSPCs. Based on a digital cytometry (d-cyto) analysis using single-cell transcriptional profiling, 85% of PB-HSPCs are shown to be CD44^+^GATA1^−^ (Fig. 3a, b). In contrast, only 6.2% CD43^+^ hESCs-HPCs were CD44^+^GATA1^−^ (Fig. 3a, b). These findings indicate that CD44 might be a previously un-recognized surface marker to label definitive multi-potent HSPCs in differentiation. We then performed flow cytometry analysis (FACS) to examine CD44 expression in a different batch of Lin^−^CD34^+^CD38^−^ PB-HSPCs as well as hESCs-derived HPCs. Consistent with d-cyto data, near 100% of fresh Lin^−^CD34^+^CD38^−^ PB-HSPCs were CD44 positive, whereas only around 50% of CD43^+^ hESCs-HPCs were CD44 positive (Fig. 3c). We then analyzed the multi-potency of CD44^+^ hESCs-HPCs in more detail through both in vitro and in vivo approaches (Fig. 3d). Firstly, based on methylcellulose (MC) colony-forming assays (MC-CFUs), the FACS-sorted CD43^+^CD44^+^ hESCs-HPCs showed significantly higher CFU efficiencies than the CD43^+^CD44^−^ cells (Fig. 3e‒g). Moreover, CFUs formed by CD43^+^CD44^−^ cells were very small and mainly erythroid ones (Fig. 3e‒g). In contrast, CD43^+^CD44^+^ cells formed big CFUs with various subtypes including CFU-E, CFU-G (granulocytes), CFU-M (macrophages) and many CFU-Mix subtypes (Fig. 3e‒g). Since the standard MC-CFU assays are less efficient for detecting myeloid and lymphoid potential at the same time, we then examined CD43^+^CD44^+^ cells through co-culturing with MS-5 stromal cells, which support the development of both human myeloid and lymphoid in vitro^6,13^. Upon co-culturing with MS-5 cells in the presence of cytokines, CD43^+^CD44^+^ cells gave rise to significant amount of myeloid cells as detected by CD15^+^ (granulocytes) and CD14^+^ (macrophages) (Fig. 3h). Moreover, we could detect a significant megakaryocyte-erythroid (Mk-Er) population in CD43^+^CD44^+^ cells co-culturing with MS5 (Fig. 3h),consistent with the recent report that the Mk-Er lineage could directly differentiate from human adult HSPCs^8^. More importantly, we detected significant CD56^+^ NK cells in the differentiation (Fig. 3h), indicating CD43^+^CD44^+^ cells hold lymphoid potential. Furthermore, through OP9-DL4 stromal cell co-culturing that supports T cell differentiation, we detected CD4 and/or CD8 positive T cells derived from the CD43^+^CD44^+^ cells (Fig. 3h). Together, these data demonstrate that CD43^+^CD44^+^ labels definitive multipotent HSPCs in hESC differentiation (hESCs-HSPCs).

### Clonal assays of lineage potential of definitive hESC-HSPCs

**Fig. 4:**
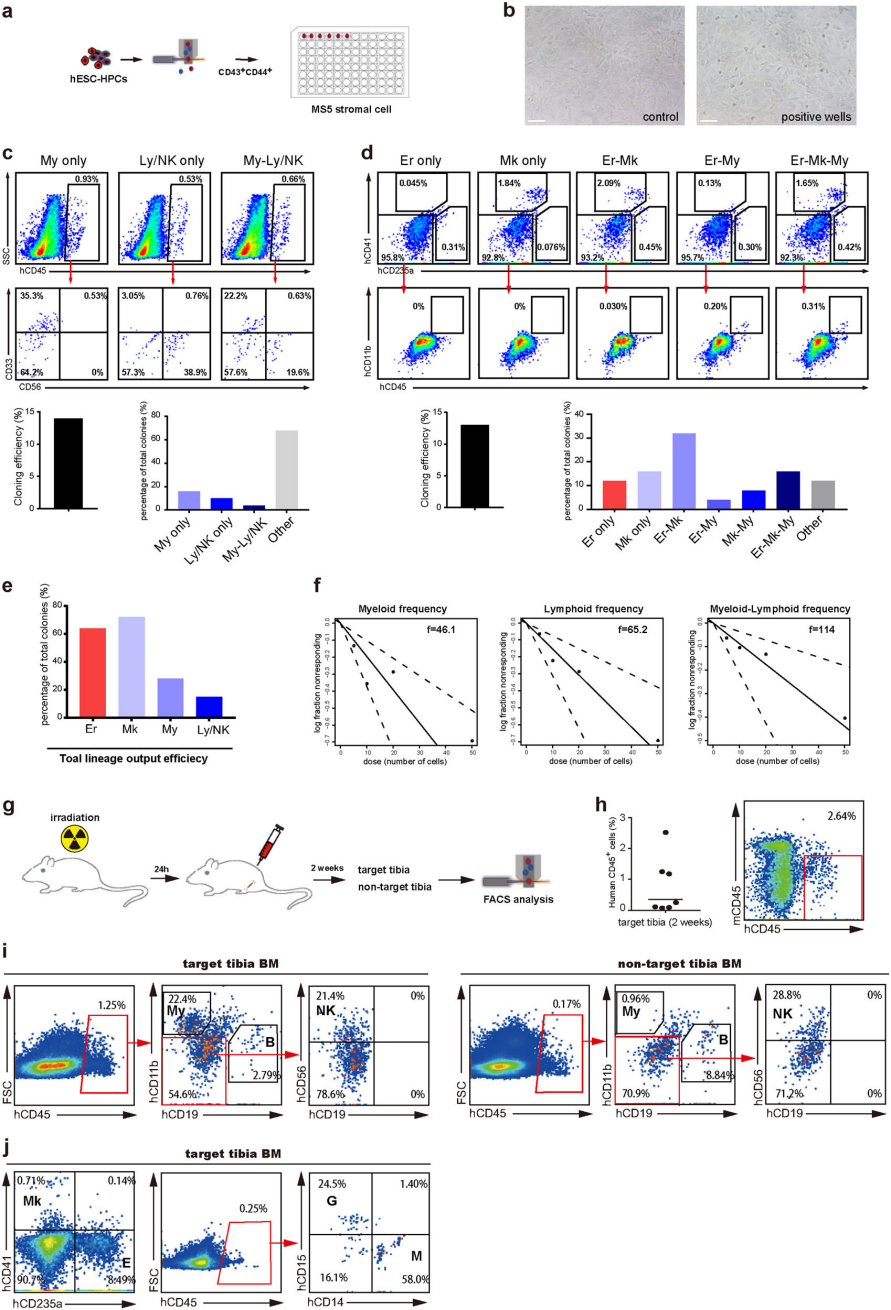
Analysis of multi-potency of hESC-HSPCs by single-cell clonal assay and in vivo transplantation. **a** Experimental design of clonal assays of lineage potential of hESC-HSPCs co-culturing with MS-5. CD43^+^CD44^+^ hESC-HSPCs were sorted at differentiation day 8 and seeded on MS-5 cells in 10 96-wells plates at single-cell level. Myeloid and/or lymphoid lineage outputs were examined 4 weeks after co-culturing. **b** Morphology of single hESC-HSPCs co-culturing with MS-5 for 4 weeks. Scale bar: 100 μm. **c** FACS analysis of myeloid and/or lymphoid lineage outputs of single hESC-HSPCs co-culturing with MS-5. The CD45^+^ human hematopoietic cells were gated 4 weeks after differentiation and analyzed for known lineage markers as indicated. CD33 for myeloid and CD56 for lymphoid/NK. Left lower panel: quantitative data of total cloning efficiency of single hESC-HSPCs that can form blood clones. Right lower panel: percentage of myeloid and/or lymphoid clones of total formed blood clones by single hESC-HSPCs. **d** FACS analysis of erythroid, megakaryocyte and/or myeloid lineage outputs of single hESC-HSPCs co-culturing with MS-5. The cells were gated 4 weeks after differentiation and erythroid clones were defined as hCD235a^+^ only (Er only). Megakaryocyte clones were defined as hCD41^+^ only (Mk only). Erythroid/megakaryocyte clones were defined as hCD235a^+^ and hCD41^+^ but negative for hCD11b (Er/Mk). Erythroid/myeloid clones were defined as hCD235a^+^ and hCD11b^+^ but negative for hCD41 (Er/My). Erythroid/megakaryocyte/ myeloid clones were defined as hCD235a^+^, hCD41^+^ and hCD11b^+^ (Er/Mk/My). Left lower panel: quantitative data of total cloning efficiency of single hESC-HSPCs that can form blood clones. Right lower panel: percentage of erythroid, megakaryocyte, and/or myeloid clones of total formed blood clones by single hESC-HSPCs. **e** Lineage cloning efficiency of single hESC-HSPCs shown in **c** and **d**, presented as the proportion of cells of various lineages in positive wells. Er, Erythroid potential (erythroid plus mixed); MK, Megakaryocyte potential (megakaryocyte plus mixed); My, Myeloid potential (myeloid plus mixed); Ly/NK, Lymphoid potential (lymphoid plus mixed). **f** Limiting-dilution assays for myeloid and/or lymphoid frequencies of hESC-HSPCs. LDA plots show the frequency (f: 1 in X cells can give rise to) of CD44^+^ hESC-HSPCs in myeloid, lymphoid and myeloid-lymphoid potential. Plots are generated by ELDA software. **g** Experimental design of in vivo transplantation of hESC-HSPCs. **h** Human CD45^+^ cell engraftment in the injected tibias of NSI mice 2 weeks after transplantation of hESC-HSPCs. **i**, **j** In vivo lineage potential of hESC-HSPCs. CD43^+^CD44^+^ hESC-HSPCs were sorted at differentiation day 8 and transplanted into the tibia of NSI mice. Multilineage outputs were examined 2 weeks after transplantation. Myeloids (My), Nature killer cells (NK), B cells, Granulocytes (G) and macrophages (M) were gated for human CD45^+^ events. Megakaryocytes and erythrocytes were defined as hCD41^+^ only (Mk) or hCD235a^+^ (E) only.

Examining lineage fate potential at the single-cell level is a golden standard to assess the multi-potency of HSPCs. We then investigated the lineage potential of CD44^+^ hESC-HSPCs at single-cell level through MS-5 co-culturing ^6,13^(Fig. 4a, b). The clonal outputs of major lineages including myeloid (My), lymphoid (Ly/NK), erythroid-megakaryocyte (Er-Mk) were analyzed based on known surface markers (Fig. 4c, d). Single CD43^+^CD44^+^ hESCs-HSPCs seeded in this condition yielded uni- and multi-potential blood colonies with the overall output clonal efficiency around 15% (Fig. 4c, d). Specifically, the percentage of uni-lineage potential for each individual blood lineage (Er only, My only, Mk only and Ly/NK only) was quite even, which was around 10% of total blood colonies for each (Fig. 4c, d). Strikingly, more than half of single cell-derived blood colonies were bi- and tri-lineage potential (Fig. 4c, d). Notably, the total output potential for each individual lineage such as Er, My, Mk and Ly/NK from all the uni-, bi- and tri-lineage colonies varied a lot between different lineages (Fig. 4e). More than 60% of total single hESC-HSPC-derived blood colonies contained either Er and/or Mk, whereas around 15% contained Ly/NK lineage (Fig. 4e), indicating an Er/Mk predominance. These features are quite consistent with those of human HSPCs in the early fetal liver that were recently reported to exhibit differential intrinsic lineage potential according to the gestational stage, i.e., with early Er predominance and later Ly representation^33^. Nonetheless, we demonstrate that CD44-labeled hESC-derived definitive HSPCs hold the multi-potency to give rise to various blood cells and immune cells based on single-cell clonal assays. Furthermore, limiting-dilution assays (LDA) generated a similar estimate on the lineage potential of myeloid and/or lymphoid in hESCs-HSPCs (Fig. 4f).

### Definitive hESC-HSPCs give rise to various blood and lymphoid lineages in vivo

To further examine the lineage potential in vivo, we transplanted FACS-sorted CD43^+^CD44^+^ hESCs-HSPCs directly into the tibia of irradiated NSI mice (Fig. 4g). We detected a significant number of CD45^+^ human cells from the bone marrow (BM) of the injected tibia 2 weeks after transplantation (Fig. 4h). The engrafted CD45^+^ human cells varied from near 1% to 3% of total cells isolated from mouse tibia and contained large proportion of CD15^+^ granulocytes, CD14^+^ macrophages as well as lymphoid lineages such as CD19^+^ B cells and CD56^+^ NK cells (Fig. 4h‒j). In addition, a large number of Mk-Er lineage progenitors could also be detected in human engraftment (Fig. 4j). Interestingly, we could detect a significant number of human B and NK lineages in BM of non-injected tibia (Fig. 4h, i), indicating homing and migration of the transplanted hESC-HSPCs in vivo. Together, these data demonstrate that the CD43^+^CD44^+^ hESCs-HSPCs could give rise to multiple lineages in vivo upon transplantation into mice.

### **Discrimination of earli**est primitive and definitive hematopoiesis in hESC differentiation

**Fig. 5:**
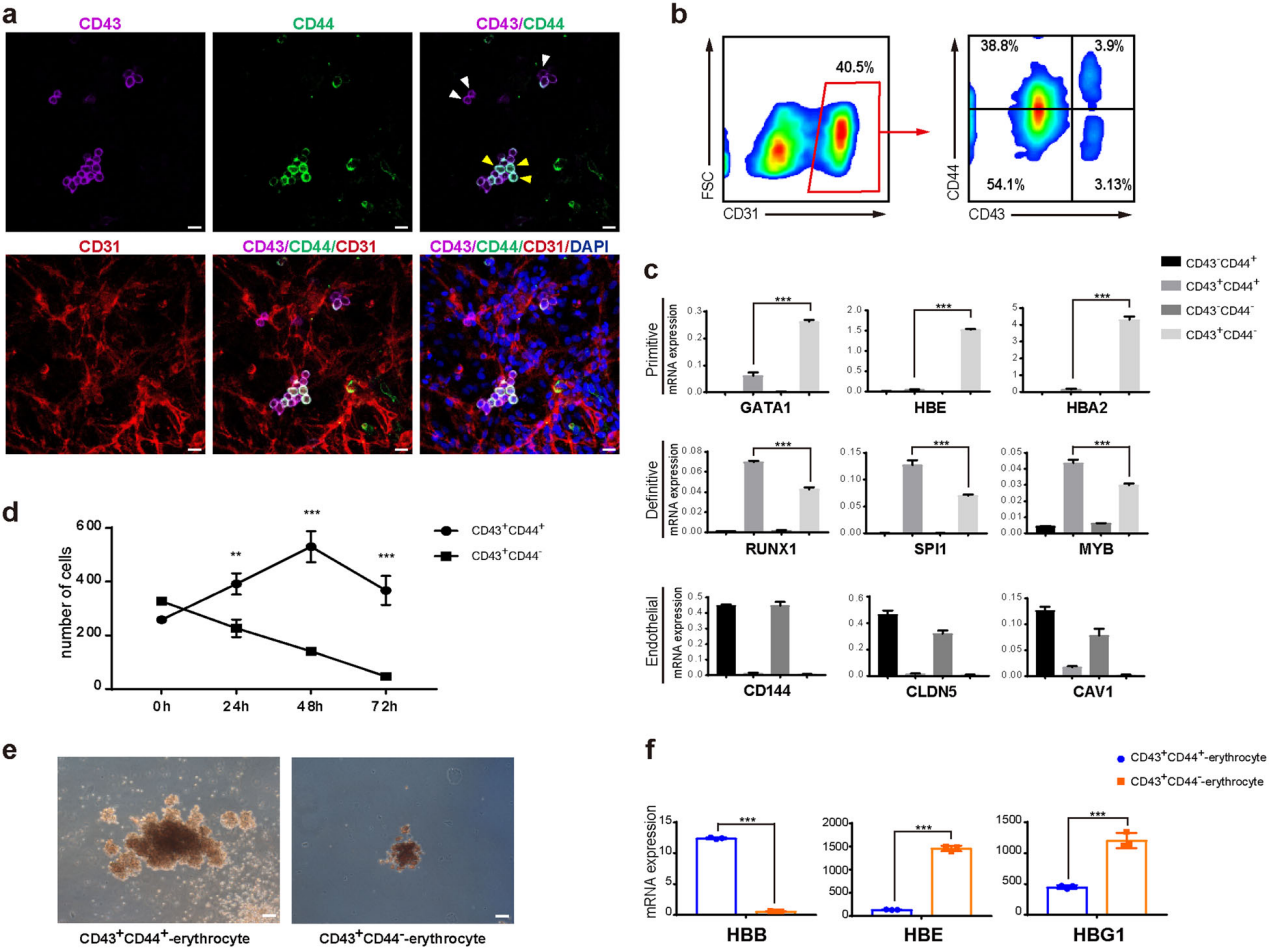
CD44 discriminates primitive and definitive hematopoiesis in hESC differentiation. **a** Immunostaining analysis of nascent HPCs derived from hESCs by anti-CD31, anti-CD43, anti-CD44 at day 8 of hematopoietic differentiation. White arrow, CD43^+^CD44^−^ cells; Yellow arrow, CD43^+^CD44^+^ cells. Scale bar, 20 μm. **b** FACS analysis of the adherent hESC-HPCs differentiated at day 8 by anti-CD31, anti-CD43, anti-CD44. **c** qRT-PCR analysis of the indicated primitive, definitive and endothelial genes in each subpopulation sorted by anti-CD31, anti-CD43, and anti-CD44 at differentiation day 8. The significance level was determined using unpaired two-tailed Student’s *t*-tests, ***P < 0.001. The data represent mean ± SD from three independent replicates (*n* = 3). **d** The expanding of CD43^+^CD44^+^ and CD43^+^CD44^-^ HPCs. The cells were sorted at differentiation day 8 and cultured in human HSC medium. The total cell numbers of the indicated population were counted at indicated time points. Significance level was determined using unpaired two-tailed Student’s *t*-tests, **P < 0.01, ***P < 0.001. The data represent mean ± SD from three independent replicates (*n* = 3). **e** Morphology of CFU-E derived from CD43^+^CD44^+^ or CD43^+^CD44^−^ HPCs. Scale bar, 100 μm. **f** qRT-PCR analysis of indicated globin genes in erythrocytes derived from CD43^+^CD44^+^ or CD43^+^CD44^−^ HPCs. The significance level was determined using unpaired two-tailed Student’s *t*-tests, ***P < 0.001. The data represent mean ± SD from three independent replicates (*n* = 3).

As reported by other groups, both the primitive and definitive hematopoiesis occur in hPSC differentiation^20,21^, but remain poorly defined due to the limited reliable markers. To track the origin of the definitive CD44^+^ hESCs-HSPCs, we analyzed the EHT in our monolayer hematopoietic differentiation. To identify cells that were undergoing EHT, we co-stained the differentiated cells at day 8 for demonstrating the endothelia cell marker, CD31, and the hematopoietic cell markers, CD43 as well as CD44. Consistent with previous reports, most round but attached cells were co-stained for both CD31 and CD43 (Fig. 5a), showing the typical phenotype of cells undergoing EHT. However, a significant number of the nascent CD43^+^ cells undergoing EHT were negative for CD44 (Fig. 5a). The EHT cells were then analyzed by FACS (Fig. 5b). The CD31-gated cells were clearly separated into 4 distinct groups based on CD44 and/or CD43 staining: CD31^+^CD43^−^CD44^−^, CD31^+^CD43^−^CD44^+^, CD31^+^CD43^+^CD44^+^ and CD31^+^CD43^+^CD44^−^. (Fig. 5b). Two cell groups with EHT phenotype, CD31^+^CD43^+^CD44^+^ and CD31^+^CD43^+^CD44^−^ showed distinct expressions of hematopoiesis related genes (Fig. 5c). Strikingly, the primitive hematopoiesis genes such as *GATA1*, *HBA2* and *HBE* were highly expressed in CD31^+^CD43^+^CD44^−^ cells, whereas they were not expressed or expressed at low levels in CD31^+^CD43^+^CD44^+^ cells (Fig. 5c). In contrast, the expressions of HSC-related genes like *RUNX1*, *MYB*, and *SPI1* were much higher in CD31^+^CD43^+^CD44^+^ cells than those in CD31^+^CD43^+^CD44^−^ cells (Fig. 5c). These data demonstrate that CD44 efficiently discriminates definitive hematopoiesis from the primitive ones at the earliest stage of human hematopoiesis. As expected, the CD43^-^ cells, either CD44^+^ or CD44^-^, expressed high endothelia genes such as *CD144*, *CLDN5*, and *CAV1* (Fig. 5c), consistent with their endothelia phenotype. Furthermore, the sorted CD44^+^ definitive HSPCs showed much better expansion than the CD44^−^ primitive progenitors in typical medium supporting human HSPCs (Fig. 5d). Functionally, CD44^+^ HSPCs could give rise to big CFU-E as well as many other lineages, whereas CD44^−^ cells only produced small CFU-E and very little myeloid cells (Fig. 5e). Furthermore, the adult globin gene like *HBB* was highly expressed in CD44^+^ HSPCs-derived erythroid cells but not in CD44^−^ cells-derived erythrocytes (Fig. 5f). In contrast, the embryonic globin genes such as *HBE* and *HBG1*, were highly expressed in CD44^−^ cells-derived erythroid cells, but their expressions were much lower in CD44^+^ cells-derived ones (Fig. 5f). Together, all these data demonstrate that CD44 expression discriminates the earliest definitive and primitive hematopoiesis in hESC differentiation.

### Human definitive and primitive hematopoiesis in early embryonic development

**Fig. 6:**
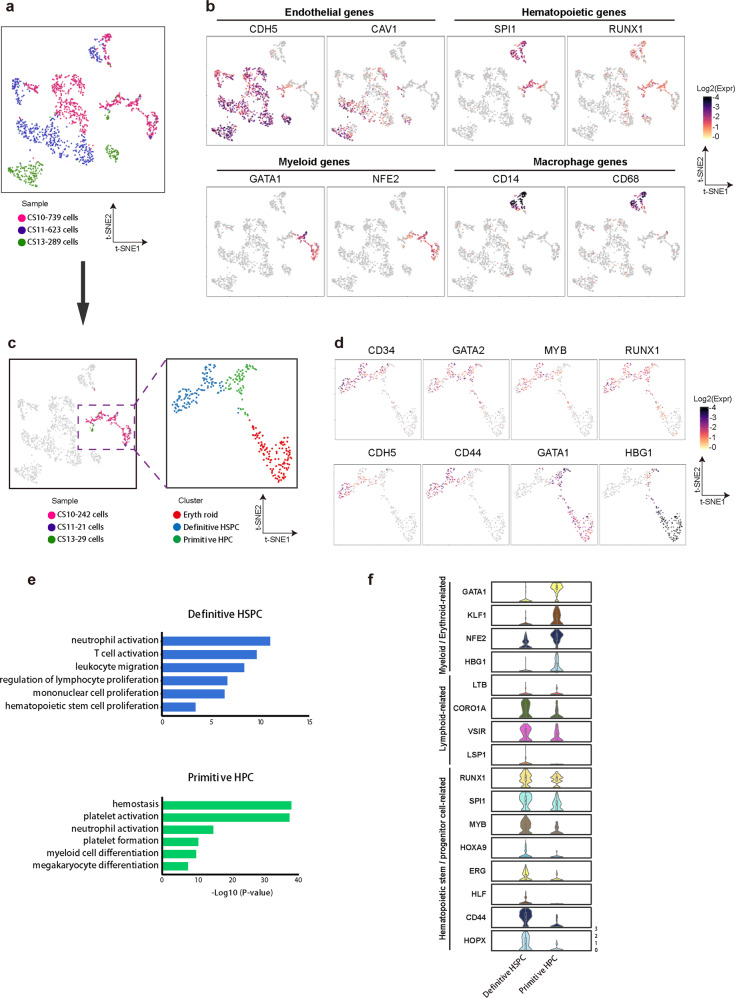
Definitive human hematopoiesis in early embryonic development. **a** *t*-SNE projection of endothelial and hematopoietic cells, resulting from sub-dividing the cells in Supplementary Fig. S1a as indicated in Supplementary Fig. S1c, assigned based on samples. Each dot represents one cell and colors represent cell samples. The legend shows the number of cells in each sample. **b** Expressions of known blood and endothelial marker genes at single-cell resolution. Color displays expression level (TPM, Log-scaled). **c** *t*-SNE projection of erythroid, definitive HSPC and primitive HPC cluster cells, resulting from sub-dividing the cells in Fig. 6a as indicated in the left panel, according to blood and endothelial marker gene expression as indicated in Fig. 6b. **d** Expression profiles of selected feature genes in erythroid, definitive HSPC and primitive HPC cluster cells. Each dot represents one cell and color displays expression level (TPM, Log-scaled). **e** Top Gene Ontology (GO) terms enriched in definitive HSPC and primitive HPC clusters. Colors represent cell clusters. **f** Violin plots show expression levels (TPM, Log-scaled) of selected myeloid/erythroid, lymphoid and hematopoietic stem/progenitor cell marker genes in definitive HSPC and primitive HPC clusters.

Definitive and primitive hematopoiesis have so far not been well characterized in human early embryonic development. To address this issue, we analyzed single-cell transcriptional profiling generated from early human embryonic samples at Carnegie stage 10–13(CS10–13), 23–30 days post coitus (dpc)^34^ (Fig. 6, Supplementary Fig. S1). Based on the expression of known endothelia and hematopoietic marker genes, such as *SPI1*, *CD34*, *RUNX1*, *CDH5*, we extracted endothelia and hematopoietic cells from early human embryonic samples (Fig. 6a, Supplementary Fig. S1). We identified a cluster of cells co-expressing both endothelia and hematopoietic genes, indicating they are nascent hematopoietic cells in the human early embryo (Fig. 6b, c). These cells were further divided into three distinct clusters based on their transcriptional profiling (Fig. 6c). One cluster is obviously the primed erythroid fate as the cells highly expressed primitive erythroid genes such as *GYPA*, *HBG1*. (Fig. 6d). The other two clusters are nascent low-primed HSPCs as they express HSPC-related genes like *CD34*, *GATA2*, *MYB*, *RUNX1*, and/or *CDH5*, the endothelia gene (Fig. 6d). Strikingly, CD44 and GATA1 showed clearly mutually exclusive expressions in two groups of low-primed HSPCs in the human embryo (Fig. 6d). Genes highly expressed in CD44^+^ group cells were significantly enriched for functions related to HSC regulation and particularly the lymphoid potential (Fig. 6e), demonstrating these cells are nascent definitive HSPCs in human early development. In contrast, genes in CD44^-^ group cells were related to Er/My fate, whereas they showed no lymphoid related function, suggesting this group of cells might be mainly primitive Er/My progenitors in human development. Indeed, selected Er/My and HSPC genes showed distinct expression patterns in CD44^+^ definitive and CD44^−^ primitive cells (Fig. 6f). Particularly, lymphoid genes were significantly enriched in CD44^+^ definitive HSPCs compared with the CD44^−^ primitive ones (Fig. 6f). Together, these data suggest that the earliest definitive and primitive hematopoiesis in human early embryonic development exhibit differential CD44 expression patterns.

### ROCK-inhibition promotes human definitive hematopoiesis

**Fig. 7:**
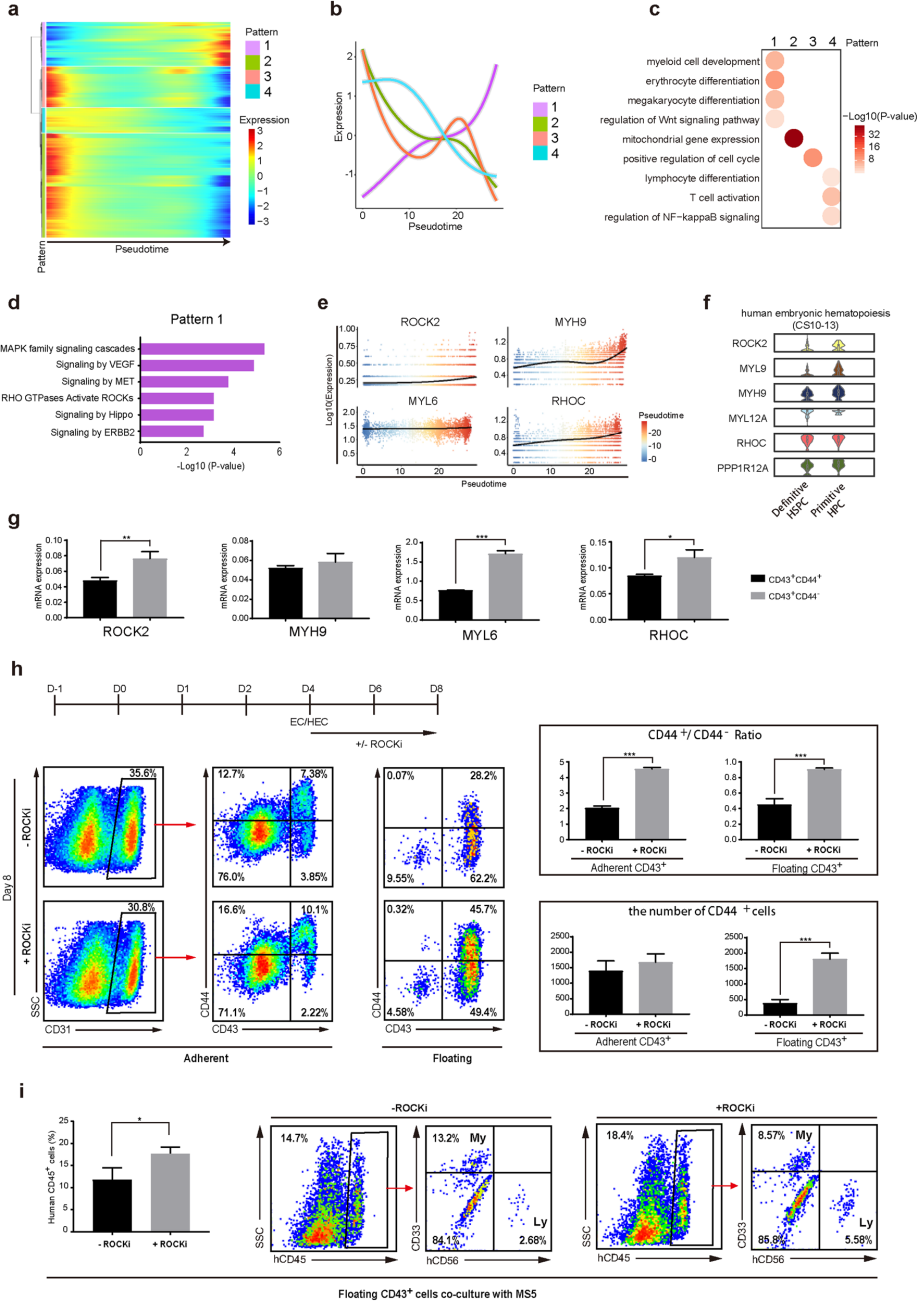
ROCK inhibition promotes the generation of definitive HSPCs from hESCs. **a**, **b** Expression profiles of genes significantly upregulated or downregulated along the pseudotime progression. Based on changing profile, genes were clustered hierarchically into four different patterns (**b**). Lines show the mean values of scaled expression levels (TPM, z-normalized) in each pattern. **c** Featured GO terms enriched in genes of each pattern. Color displays *P*-value (Log-scaled) of GO terms. **d** KEGG enriched genes in pattern 1 indicated in **b**. **e** Expression profiles of ROCK pathway-related genes along the pseudotime. Each dot represents one cell and color represents pseudotime value. Line shows the mean expression level (TPM, Log-scaled). **f** Violin plots show expression levels (TPM, Log-scaled) of these ROCK signaling genes in early definitive and primitive HSPCs identified in human embryo. **g** qRT-PCR analysis of the ROCK pathway-related genes in CD43^+^CD44^+^ or CD43^+^CD44^−^ HPCs sorted at differentiation day 8. The significance level was determined using unpaired two-tailed Student’s *t*-tests, *P < 0.05, **P < 0.01, ***P < 0.001. The data represent mean ± SD from three independent replicates (*n* = 3). **h** Analysis of ROCK inhibition in hESC differentiation. The chemical inhibitor, Y-27632, was added in EHT medium at differentiation day 4 as indicated. The adherent or floating cells at differentiation day 8 were analyzed by FACS for the expression of CD31, CD43, and CD44. Right panel: quantitative data of each indicated population at differentiation day 8 of hESCs. The significance level was determined using unpaired two-tailed Student’s *t*-tests, ***P < 0.001. The data represent the mean ± SD from three independent replicates (*n* = 3). **i** Function Analysis of CD43^+^ HSPCs generated under ROCKi treatment. Y-27632 was added in EHT medium at differentiation day 4 as indicated. The floating CD43^+^ cells at differentiation day 8 were counted and co-cultured with MS5 with 10,000 cells per well. Human CD45^+^ cells were gated 2 weeks after differentiation and analyzed for the known lineage markers as indicated. My, myeloid; Ly, lymphoid. The significance level was determined using unpaired two-tailed Student’s *t*-tests, *P < 0.05. The data represent mean ± SD from three independent replicates (*n* = 3).

The identification of CD44 to discriminate the definitive and the primitive hematopoiesis allows us to investigate pathways to regulate these two functionally distinct waves of hematopoiesis. Based on the selected marker genes, the pseudotime progression generated from all the CD43^+^ hESCs-derived cells represent a fate change from a low-primed definitive state to primitive erythroid fate (Fig. 2b). We, therefore, characterized dynamic genes along with the pseudotime progression (Fig. 7a, b). Consistently, genes with their expression increased along pseudotime are highly related to myelo-erythroid fate while those downregulated genes are related to lymphocyte fate regulation, cell cycle, etc. (Fig. 7c), reflecting a gradual definitive to primitive fate change. We identified signaling pathways that were differentially regulated between human primitive and definitive hematopoiesis (Fig. 7d). Many of these pathways are known to play important roles in hematopoiesis, such as MAPK, VEGF^35–38^ (Fig. 7d). We noted that ROCK pathway genes showed an obvious upregulation in fate change from definitive to primitive hematopoiesis (Fig. 7d, e), but their roles in human hematopoiesis has not been recognized before. Interestingly, these ROCK signaling genes also exhibited differential expression in early definitive and primitive HSPCs identified in the human embryo (Figs. 6, 7f). We further confirmed that the expressions of these ROCK pathway genes were significantly higher in CD43^+^CD44^−^ primitive progenitors than those in CD43^+^CD44^+^ definitive ones in hESC differentiation (Fig. 7g). These data indicate that ROCK signaling positively regulates early primitive hematopoiesis in humans. To further examine the role of ROCK in human hematopoiesis, we added the known chemical inhibitor for ROCK (Y-27632) during EHT process in hESC differentiation (Fig. 7h). Indeed, the percentage of definitive progenitors that were either undergoing EHT (CD31^+^CD43^+^CD44^+^) or became floating after EHT (CD43^+^CD44^+^) significantly increased in condition with ROCK inhibition (ROCKi) (Fig. 7h). Notably, the total number of floating definitive progenitors was also significantly higher in the presence of ROCK inhibitors (right lower panel, Fig. 7h). Functionally, CD43^+^ HSPCs generated under ROCKi treatment produced more human CD45^+^ cells containing both myeloid and lymphoid cells upon co-culturing with MS5 (Fig. 7i). In all, we reveal that ROCK inhibition greatly promotes human definitive hematopoiesis to generate multipotent progenitors in hPSC differentiation.

## Discussion

Given the therapeutic potential of blood and the immune cells such as NK and T cells, the generation of definitive multi-potent HSPCs in vitro, particularly from human PSCs has been a long-sought goal. However, HPCs derived from hPSCs have very limited potential in generating multiple blood lineages, particularly a very limited lymphoid potential^23,24,27,28^. During early hematopoietic development, the lymphoid lineages are mainly generated through definitive hematopoiesis^14–16^. Limited knowledge on the characterization of human early definitive hematopoiesis largely impedes the progress to generate definitive HSPCs in vitro. Here, we resolved cell populations and characterized the human definitive and primitive hematopoiesis at the earliest hematopoiesis both in hESC differentiation and early embryonic development. We identified CD44 to discriminate two distinct hematopoiesis at the earliest stage of human hematopoietic development, which allows us to generate definitive HSPCs through hPSC differentiation. Functionally, the generated definitive HSPCs from hESCs hold the multi-potency to give rise to various blood cells such as Er/Mk, My, and more importantly the immune cells such as NK, T cells, as validated by single-cell assays. Strikingly, these hESCs-derived definitive HSPCs exhibit homing and migration and contribute multiple lineages in vivo upon transplantation, including My, Mk-Er, and the lymphoid lineages such as NK and B cells, thus supporting their therapeutic potential.

Notably, the definitive HSPCs derived from hESCs largely resemble human early embryonic HSPCs in terms of intrinsic lineage potential, with Er/My/Mk predominance and lower Ly representation (Fig. 4)^33^. This consistency supports our findings as a valuable model to investigate human early hematopoiesis. Indeed, the earliest primitive and definitive hematopoiesis at EHT displays highly similar phenotype in hESC differentiation and human embryo (Figs. 5, 6). Furthermore, we characterized dynamic gene expressions in human definitive and primitive hematopoiesis and identified signaling pathways that differentially regulate definitive and primitive hematopoiesis^35–38^ (Fig. 7). Among these pathways, ROCK signaling has not been known to regulate human hematopoietic development. Strikingly, inhibition of ROCK greatly enhances human definitive hematopoiesis in hESC differentiation (Fig. 7). We thus established an enhanced approach to generate and then isolate multipotent definitive HSPCs from human PSCs. We lastly confirmed that CD44-labeled definitive HSPCs could also be generated from human urine cell-derived iPSCs (Supplementary Fig. S2). Thus, our studies not only provide a valuable model to understand human hematopoietic development and also a firm basis to generate patient’s specific blood and immune cells for clinical purposes.

## Materials and methods

### hPSCs culture and maintenance

All human PSC cell lines were maintained on Matrigel (1:100 dilution; BD)-coated plates in mTeSR1 medium (Stem Cell Technologies) supplemented with 1% penicillin-streptomycin (Hyclone). Medium was changed every day and cells were passaged 1:3 onto fresh Matrigel-coated plates every 3 days using 0.5 mM ethylenediaminetetraacetic acid disodium salt (EDTA-2Na). All of the human PSC cell lines mentioned above were maintained at 37 °C, 20% O_2_ and 5% CO_2_ condition and had been tested to be free of mycoplasma contamination.

### Hematopoietic differentiation of hPSCs

Prior to differentiation, the hPSCs should be 80%~90% confluent and were dissociated into single cells using Accutase (Sigma). And then cells were plated onto Growth Factor Reduced (GFR) Matrigel (1:100 dilution; BD)-coated six-well plates at a proper initial density of about 3 × 10^5^/well. Especially, in order to inhibit hPSCs apoptosis, thiazovivin (0.1 μM, Selleck) was added in the medium. After 24‒36 h culture, the cells were about 10% confluent and this day was designated as day 0 (D0). Then, the hPSCs were induced for stepwise differentiation in basal medium (BM) supplemented with cytokines and inhibitors in the following days. D0‒D1: 40 ng/ml BMP4 (Peprotech), 30 ng/ml ACTIVIN A (Sino Biological), 20 ng/ml bFGF (Sino Biological), 6 μM CHIR99021 (Selleck) and 10 μM LY294002 (Selleck); D1‒D2: 30 ng/ml BMP4, 1 μM A8301 (Selleck) and 2 μM IWR-1-endo (Selleck); D2‒D4: 40 ng/ml VEGF (Sino Biological) and 50 ng/ml bFGF; D4‒D8: 40 ng/ml VEGF, 50 ng/ml bFGF, 10 μM SB431542 (Selleck), 10 ng/ml SCF (Peprotech), 50 ng/ml TPO (Sino Biological), 10 ng/ml IL3 (Sino Biological), 50 ng/ml IL6 (Sino Biological) and 50 ng/ml FLT3L (Peprotech). BM: DMEM/F12 (GIBCO) + 1% penicillin-streptomycin (Hyclone) + 1% insulin-transferrin-selenium (ITS, GIBCO) + 70 μg/ml vitamin C (Vc, 2-Phospho-L-ascorbic acid trisodium salt solution, Sigma). Particularly, the osmotic pressure of the HDM was adjusted by 9% NaCl to about 340. The hematopoietic differentiation medium in each step should be changed every day and the differentiating cells were differentiated in 37 °C, 20% O_2_ and 5% CO_2_ condition.

### Quantitative real-time PCR (qRT-PCR)

The total RNA was extracted from cells using the RaPure Total RNA Micro Kit (Magen) and 2 μg RNA was reversely transcribed into cDNA with a HiScript II 1st Strand cDNA Synthesis Kit (Vazyme). Then, qRT-PCR was performed with ChamQ SYBR qPCR Master Mix (Vazyme) and a CFX96 machine (Bio-Rad). GAPDH was used for normalization. All data were analyzed with 3 replicates and all primers used in this study were listed in Supplementary Table S1.

### CFU assay and cell morphology

The CFU assay was performed according to the manufacturer’s instruction of Methocult H4435 (Stem Cell Technologies). Firstly, an indicated number of single cells were suspended into 120 μl IMDM medium supplemented with 2% FBS (Biological Industries), and then cell suspension was added into 1 ml Methocult H4435. Next, we transferred the mixture to 35 mm ultra-low attachment plates (Stem Cell Technologies) and rotated the plates gently to spread methylcellulose medium over the surface of the dish. We placed three dishes within a 100 mm petri dish containing 3 mL sterile water and incubated the dishes in 37 °C, 20% O_2_ and 5% CO_2_ condition. The CFUs were classified and calculated according to the morphology after 2 weeks. All data were analyzed with three replicates. Then, we harvested the proper number of cells from different colony types in CFU assay, rinsed them twice with DPBS (gibco) and resuspended the cells in 200 μl DPBS. The morphology of the cell was showed by microscopy after cytospin preparation and staining with May–Grunwald–Giemsa.

### Flow cytometry and cell sorting

A single-cell suspension was prepared by Accutase (Sigma) and filtered through 70 μm filter. Then, cells were stained by multicolor antibody combinations in DPBS supplemented with 2% FBS and incubated at 4 °C for 20–30 min. The cells were sorted by the Aria II (BD) or Moflo (Beckman). Antibodies were listed in Supplementary Table S2.

### In vitro lympho-myeloid lineage differentiation at bulk

MS-5 cells were seeded onto 0.1% gelatin-coated 24-well plate at an initiating density of 2 × 10^4^/well in α-MEM medium (Thermo Fisher) supplemented with 10% FBS (Gibco), 1% penicillin-streptomycin (Hyclone) and 1% GlutaMAX^TM^ (Gibco). Twenty-four hours after plating of MS-5 stroma, 1 × 10^4^ cells were added into each well in the presence of 0.1 μM DuP-697 (Biovision), 20 ng/ml SCF (Peprotech), 10 ng/ml G-CSF (Peprotech), 10 ng/ml FLT3L (Peprotech), 10 ng/ml IL-2 (Peprotech), and 10 ng/ml IL-15 (Sino Biological). Half of the medium was changed twice every week and cocultures were transferred onto fresh MS-5 stroma every two weeks through 40 μm filter to remove the stromal cells. All of the cells in each well were harvested and analyzed by flow cytometry at week 1 and week 4. The antibodies used to read the lineage outputs in this assay were anti-human CD45-PE, anti-human CD14-PE-Cy7, anti-human CD15-APC, anti-human CD19-FITC, anti-human CD56-PerCP-Cy5.5, anti-human CD45-APC, anti-human CD235a-FITC, and anti-human CD41-PE.

### In vitro lympho-myeloid lineage differentiation at single-cell level

For single-cell analysis, MS-5 cells were seeded onto 0.1% gelatin-coated 96-well plate at an initiating density of 2.5 × 10^3^/well in α-MEM medium (Thermo Fisher) supplemented with 10% FBS (Gibco), 1% penicillin-streptomycin (Hyclone), 1% GlutaMAX^TM^ (Gibco), 0.1 μM DuP-697 (Biovision), 20 ng/ml SCF (Peprotech), 10 ng/ml G-CSF (Peprotech), 10 ng/ml FLT3L (Peprotech), 10 ng/ml IL-2 (Peprotech) and 10 ng/ml IL-15 (Sino Biological). Twenty-four hours later, single CD44-positive hESC-HPCs were sorted into 96-well plates and half of the medium was changed every week. After culturing for 3‒4 weeks, all of the cells in each well were resuspended by physical dissociation and analyzed by flow cytometry, and wells with more than 15 human CD235a^+^, CD41^+^, CD33^+^/CD11b^+^, or CD56^+^ cells were considered positive. The antibodies used to read the lineage outputs in this assay were anti-human CD45-PE-Cy7, anti-human CD33-PE, anti-human CD56-APC.

### In vitro T lineage differentiation

OP9-hDL4 cells were seeded onto a 24-well plate coated by 0.1% gelatin at a density of 2 × 10^4^/well in α-MEM medium (Thermo Fisher) supplemented with 20% FBS (Gibco), 1% penicillin-streptomycin (Hyclone) and 1% GlutaMAX^TM^ (Gibco). One day after plating of OP9-hDL4 cells, 1×10^4^ cells were deposited into each well containing 10 ng/ml SCF (Peprotech), 5 ng/ml FLT3L (Peprotech) and 5 ng/ml IL-7 (Sino Biological). Half of the medium was changed twice every week. Harvested cells in each well were analyzed by flow cytometry at week 2 and the antibodies used to read the lineage outputs in this assay were as follows: anti-human CD45-PE, anti-human CD4-PE-Cy7, anti-human CD8-APC-Cy7.

### Mice and in vivo myeloid lineage differentiation

NSI (NOD/SCID IL2rg^−/−^) mice were purchased from Peng Li’s lab in Guangzhou Institutes of Biomedicine and Health, Chinese Academy of Sciences (GIBH, CAS). Mice were bred and housed in the SPF-grade animal care facility of the GIBH. All the animal experiments had been approved by the Animal Ethnical Committee of GIBH and performed according to the ISSCR guidelines. Mice (6- to 8-week old) were sublethally irradiated (1.2–1.5 Gry) 12‒24 h before transplantation. Then, mice were anesthetized by intraperitoneal injection with 2,2,2-Tribromoethanol (Sigma) and cells were transplanted intratibially into the mice. In brief, an insulin needle was used to drill the tibia firstly, and then a volume of 20–25 μl cells were transplanted into the tibia with another insulin needle. After two weeks, peripheral blood was isolated from the orbital venous plexus, and then mice were euthanized and the BM in tibia was flushed by PBS with a 1 ml needle. The potential contamination of mouse CD45+ cells had been excluded through strict gating using negative control. For lineage differentiation analysis, the peripheral blood and marrow were processed by ACK lysis buffer and then stained using the following antibodies: anti-human CD45-PE, anti-human CD45-PE-Cy7, anti-human CD14-PE-Cy7, anti-human CD15-APC, anti-human CD11b-APC, anti-human CD19-FITC, anti-human CD56-APC-Cy7, anti-human CD235a-PE, anti-human CD235a-FITC, anti-human CD41-PE-Cy5, and anti-human CD41-PE.

### LDA assay

For the limiting-dilution assay (LDA), different doses of CD44 positive hESC-HPCs (5, 10, 20, and 50 cells) were sorted into 96-well plates pre-plated with 2.5 × 10^3^ MS-5 cells per well supplemented with 10% FBS (Gibco), 1% penicillin-streptomycin (Hyclone), 1% GlutaMAX^TM^ (Gibco), 0.1 μM DuP-697 (Biovision), 20 ng/ml SCF (Peprotech), 10 ng/ml G-CSF (Peprotech), 10 ng/ml FLT3L (Peprotech), 10 ng/ml IL-2 (Peprotech) and 10 ng/ml IL-15 (Sino Biological). Half of the medium was changed every week. After culturing for 3‒4 weeks, all of the cells in each well were resuspended by physical dissociation and analyzed by flow cytometry. The antibodies used to read the lineage outputs in this assay were anti-human CD45-PE-Cy7, anti-human CD33-PE, anti-human CD56-APC. Frequency calculations were performed using ELDA software (http://bioinf.wehi.edu.au/software/elda/).

### Immunofluorescence

The adherent cells were fixed by 4% paraformaldehyde (PFA) for 30 min and incubated with primary antibodies overnight at 4 °C. After that, cells were washed with PBS and incubated with specific secondary antibodies. Nuclei were stained with DAPI. Stained cells were observed using a LSM800 confocal microscope (Carl Zeiss). Antibodies were listed in Supplementary Table S2.

### Single-cell RNA sequencing

Fresh mobilized PB samples were obtained as a gift from the Department of Hematology, The Third Affiliated Hospital, Sun Yat-sen University. They were processed within 24 h after received. Mononuclear cells were isolated firstly by removing of red blood cells using ACK lysis buffer, and then CD34^+^ fraction was separated by CD34 Microbead kit and magnetic-activated cell-sorting separation columns (Miltenyi Biotec) according to the manufacturer’s instruction. For cell sorting, PB CD34^+^ fraction was stained with human hematopoietic lineage FITC cocktail (CD4, CD8, CD11b, CD14, CD19, CD20, CD56, CD235a, and CD10), anti-human CD34-Percp-Cy5.5 and anti-human CD38-APC and sorted by the Aria II (BD). Human ESCs were differentiated into blood lineages in a monolayer, defined condition, and the floating blood cells at differentiation day 8 were collected and sorted by CD43. We have conducted multiple differentiation experiments and tested the CD43 expression of hESC-HPC to ensure the repeatability and consistency of the experiment, and then sorted CD43^+^ cells from one experiment for single-cell RNA sequencing. The sorted cells were analyzed by 10× genomics for single-cell RNA sequencing (scRNA-seq). The single-cell libraries were prepared following the Chromium Single Cell 3′ Reagent Kits User Guide, quantified by Quant-iT dsDNA Assay Kit with high sensitivity (Thermo Fisher) on Qubit 2.0, and in the end, sequenced on illumina NextSeq 500.

### scRNA-seq data processing

10× sequencing data were processed with cellranger (version 2.1.1) with default parameters. Then we performed quality control to filter low-quality cells. Cells with UMI counts range in Mean ± 4 SD (log transformed) were kept; cells with more than 10% of reads mapped to mitochondrial genes were removed. For hESC-derived cell samples, the Mean Reads per Cell is 83238, the Median Gene numbers per Cell is 3932, the Median UMI Counts per Cell is 25354; For the PB HSPC sample, the Mean Reads per Cell is 71890, the Median Genes per Cell is 1953, the Median UMI Counts per Cell is 7718.

### Dimension reduction and clustering

We used Seurat (version 3.0.0) to normalize data and reduce dimension. Only genes expressed in at least three cells were retained and only cells that express more than 200 genes were retained. Then, FindVariableGenes function was applied to select highly variable genes on log-transformed data. SCTransform function was used to normalize data. PCA was performed using highly variable genes. JackStraw function was used to select significant PCs, and PCs with *P*-value < 0.05 were used for downstream analyses. Cells were projected in two-dimension using RunTSNE function. In integrated analysis of PB-D8 sample, FindClusters function was used to perform clustering with parameter “resolution” = 0.04, resulting in three clusters.

### Identification of DEGs

DEGs were identified using FindMarkers function. Genes with log-fold-change more than 0.1, *P*-value < 0.01, and the difference of pct1 and pct2 more than 0.05 were selected as DEGs.

### Pseudotime trajectory analysis

Pseudotime trajectory analysis was performed with Monocle (version 2.10.1). Genes with more than fitted dispersion evaluated using dispersionTable function were identified as highly variable genes. The Discriminative Dimensionality Reduction with Trees (DDRTree) method was used to reduce dimensions. Significant DEGs along pseudotime were identified by differentialGeneTest function.

### d-cyto Analysis

d-cyto Analysis was inspired by flow cytometry. It takes RNA expression levels as inputs. The Axis panel was divided into tiles and the color of each tile represents the number of cells in it. Cells were gated depending on gene-expression levels. Percentages of cells in gate were calculated.

### GO analysis

GO enrichment was performed using clusterProfiler (version 3.10.1).

### Statistical analysis

Data were presented as mean ± SD, and statistics were determined by unpaired two-tailed Student’s *t*-test (*t*-test). *P* value < 0.05 was considered statistically significant. *P < 0.05; **P < 0.01; ***P < 0.001. No statistical method was used to pre-determine the sample size. No samples were excluded for any analysis. No randomization was used for allocating animal groups. No blinding done in animal experiments.

## Supplementary information

Figure S1

Figure S2

Table S1

Table S2

## Data Availability

The RNA-Seq data have been deposited in the Gene Expression Omnibus database under the accession code GSE148215. The authors declare that all data supporting the findings of this study are available within the article and its supplementary information files or from the corresponding author (Dr. Guangjin Pan, pan_guangjin@gibh.ac.cn) upon reasonable request.

## Supplementary information

**Supplementary Information** accompanies the paper at (10.1038/s41421-020-00213-6).
