# Supplementary material for: Characterization and generation of human definitive multipotent hematopoietic stem/progenitor cells
Source: Cell Discov. 2020 Dec 1;6:89. doi: 10.1038/s41421-020-00213-6 (PMC7705709; doi:10.1038/s41421-020-00213-6)
Supplement: Supplementary file 1 — Figure S1 [file 41421_2020_213_MOESM1_ESM.pdf]

Supplementary Figure 1

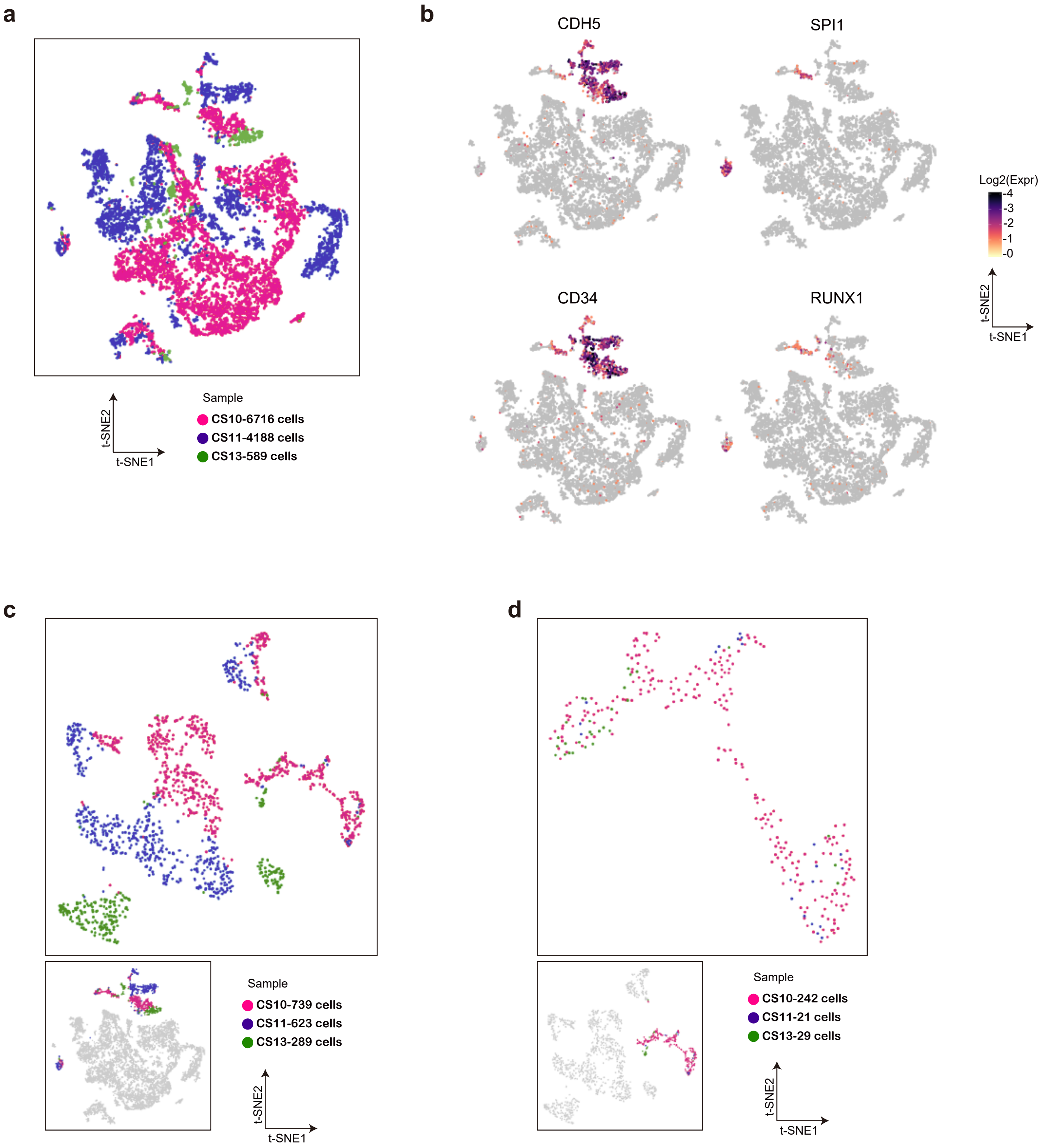

Supplementary Figure 1

a: t-SNE projection of CS10 (23 dpc), CS11 (24 dpc) and CS13 (30 dpc) cells assigned based on samples. Each dot represents one cell and colors represent cell samples. Legend shows the number of cells each sample has.

b: Expressions of endothelial and hematopoietic marker genes at single cell resolution in CS10, CS11 and CS13 samples. Color displays expression levels (TPM, log-scaled).

c: t-SNE projection of endothelial and hematopoietic cells, resulting from sub-dividing the cells in Fig.S1a as indicated in the lower left frame, assigned based on samples, according to the expression of known endothelial and hematopoietic marker genes in Fig.S1b. Legend shows the number of cells each sample has.

d: t-SNE projection of erythroid, definitive HSPC and primitive HPC cluster cells, resulting from sub-dividing the cells in Fig.S1c as indicated in the lower left frame, assigned based on samples, according to blood and endothelial marker gene expression as indicated in Fig.6b. Legend shows the number of cells each sample has.
