# Supplementary material for: Characterization and generation of human definitive multipotent hematopoietic stem/progenitor cells
Source: Cell Discov. 2020 Dec 1;6:89. doi: 10.1038/s41421-020-00213-6 (PMC7705709; doi:10.1038/s41421-020-00213-6)
Supplement: Supplementary file 2 — Figure S2 [file 41421_2020_213_MOESM2_ESM.pdf]

Supplementary Figure 2

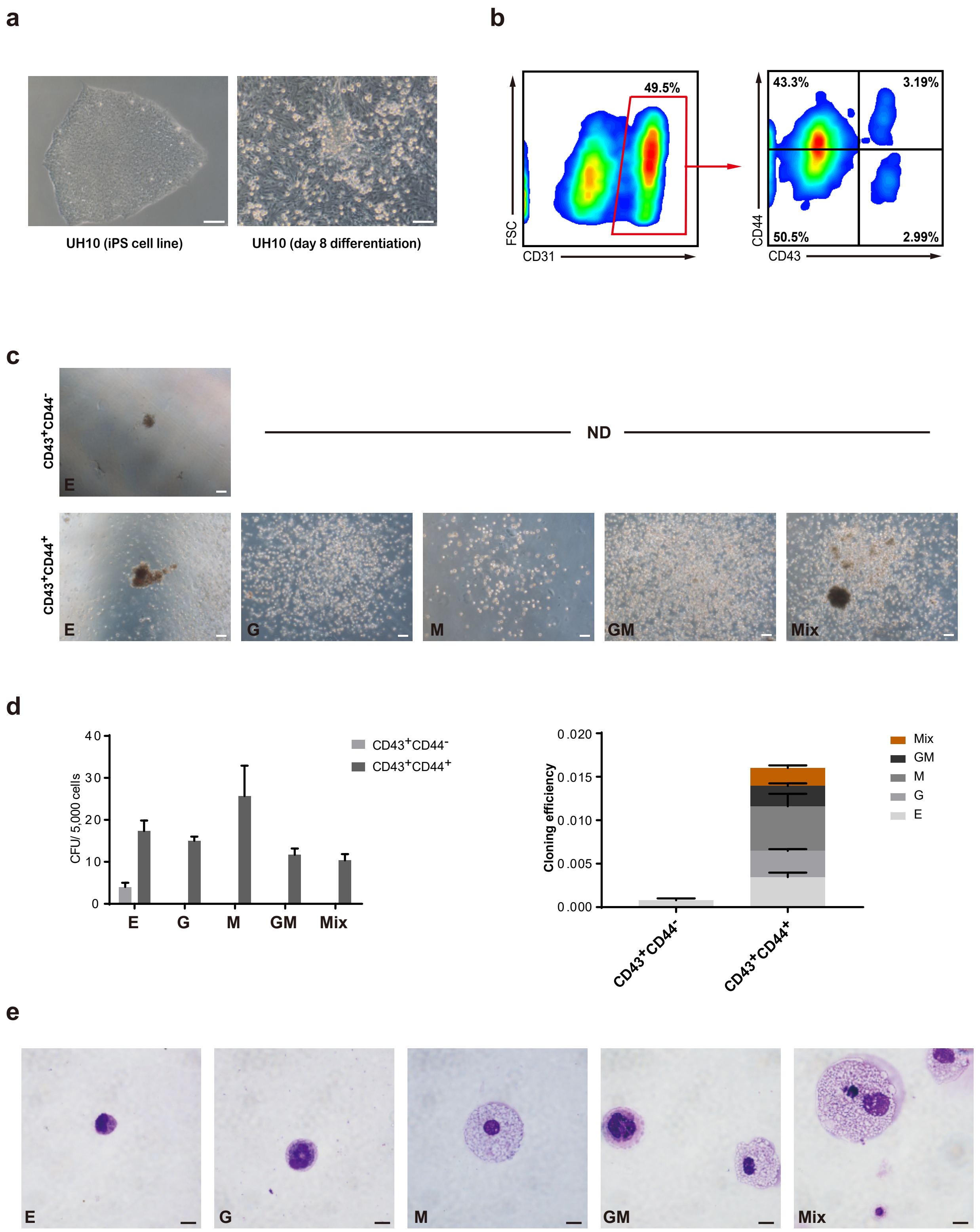

Supplementary Figure 2

a: Morphology of the UH10 (iPS cell line) under normal culture condition and at day 8 of differentiation. Scale bar: 100μm.

b: FACS analysis of the adherent iPS-HPCs differentiated at day 8 by anti-CD31, anti-CD43, anti-CD44.

c: Representative pictures of CFUs formed by iPS-HPCs sorted by $CD43^{+}CD44^{+}$ or $CD43^{+}CD44^{-}$. Scale bar: 100μm. ND: not detected. E, erythroid; G, granulocytes; M, macrophages; GM, granulocyte and monocyte-macrophage; Mix, mixed erythro-myeloid.

d: Quantitative analysis of various blood CFUs formed by the $CD43^{+}CD44^{+}$ or $CD43^{+}CD44^{-}$ iPS-HPCs sorted at day 8 differentiation.

e: May–Grunwald–Giemsa staining of different blood cells isolated from CFUs derived from $CD43^{+}CD44^{+}$ iPS-HPCs. Scale bar: 10μm.
