# Supplementary material for: Characterization and generation of human definitive multipotent hematopoietic stem/progenitor cells
Source: Cell Discov. 2020 Dec 1;6:89. doi: 10.1038/s41421-020-00213-6 (PMC7705709; doi:10.1038/s41421-020-00213-6)
Supplement: Supplementary file 3 — Table S1 [file 41421_2020_213_MOESM3_ESM.pdf]

Supplementary Table S1. Primers used in qRT-PCR

Q-H-GATA1-F	CTGTCCCCAATAGTGCTTATGG
Q-H-GATA1-R	GAATAGGCTGCTGAATTGAGGG
Q-H-HBE-F	ATGGTGCATTTTACTGCTGAGG
Q-H-HBE-R	GGGAGACGACAGGTTTCCAAA
Q-H-HBA2-F	TCTCCTGCCGACAAGACCAA
Q-H-HBA2-R	GCAGTGGCTTAGCTTGAAGTTG
Q-H-RUNX1-F	CTGCCCATCGCTTTCAAGGT
Q-H-RUNX1-R	GCCGAGTAGTTTTCATCATTGCC
Q-H-SPI1-F	GTGCCCTATGACACGGATCTA
Q-H-SPI1-R	AGTCCCAGTAATGGTCGCTAT
Q-H-MYB-F	GAGGTGGCATAACCACTTGAA
Q-H-MYB-R	AGGCAGTAGCTTTGCGATTTC
Q-H-CD144-F	GTTCACGCATCGGTTGTTCAA
Q-H-CD144-R	CGCTTCCACCACGATCTCATA
Q-H-CLDN5-F	CTCTGCTGGTTCGCCAACAT
Q-H-CLDN5-R	CAGCTCGTACTTCTGCGACA
Q-H-CAV1-F	GCGACCCTAAACACCTCAAC
Q-H-CAV1-R	ATGCCGTCAAAACTGTGTGTC
Q-H-HBB-F	AGGAGAAGTCTGCCGTTACTG
Q-H-HBB1-R	CCGAGCACTTTCTTGCCATGA
Q-H-HBG1-F	TCAAGGCACATGGCAAGA
Q-H-HBG-R	TCACCAGCACATTTCCCA
Q-H-ROCK2-F	TTGCTCTGGATGCAATACACTC
Q-H-ROCK2-R	TCTCGCCCATAGAAACCATCA
Q-H-MYH9-F	CCTCAAGGAGCGTTACTACTCA
Q-H-MYH9-R	CTGTAGGCGGTGTCTGTGAT
Q-H-MYL6-F	GAAGACCAGACCGCAGAGTTC
Q-H-MYL6-R	TCCAGCACCTTCACATTCATC
Q-H-RHOC-F	CCTGAGGCAAGACGAGCAC
Q-H-RHOC-R	GATCCGGTTCGCCATGTCC
