# Supplementary material for: Characterization and generation of human definitive multipotent hematopoietic stem/progenitor cells
Source: Cell Discov. 2020 Dec 1;6:89. doi: 10.1038/s41421-020-00213-6 (PMC7705709; doi:10.1038/s41421-020-00213-6)
Supplement: Supplementary file 4 — Table S2 [file 41421_2020_213_MOESM4_ESM.pdf]

Supplementary Table S2. Antibodies

**Antibodies**	**Vendor**	**Cat#**	**Dilution**
Anti-human CD31-PE	BD Biosciences	555446	1:100
Anti-human CD34- PerCP-Cy5.5	BD Biosciences	347203	1:100
Anti-human CD43-APC	BD Biosciences	560198	1:100
Anti-human CD44-FITC	BD Biosciences	555478	1:100
Anti-human CD44-PE-Cy7	Biolegend	103030	1:100
Anti-human CD38-APC	BD Biosciences	555462	1:100
anti-human CD45-PE	eBioScience	12-9459-42	1:100
anti-human CD14-PE-Cy7	eBioScience	45-0149-42	1:100
anti-human CD15-APC	BD Biosciences	561716	1:100
anti-human CD19-FITC	Biolegend	302206	1:100
anti-human CD56-PerCP-Cy5.5	Biolegend	318322	1:100
anti-human CD45-APC	BD Biosciences	560973	1:100
anti-human CD235a-FITC	eBioScience	11-9987-82	1:100
anti-human CD41-PE	Biolegend	303705	1:100
anti-human CD45-PE-Cy7	Biolegend	304016	1:100
anti-human CD33-PE	BD Biosciences	347787	1:100
anti-human CD56-APC	BD Biosciences	555518	1:100
anti-human CD4-PE-Cy7	BD Biosciences	560909	1:100
anti-human CD8-APC-Cy7	Biolegend	300926	1:100
anti-human CD11b-APC	elabscience	ESHF078	1:100
anti-human CD56-APC-Cy7	Biolegend	318332	1:100
anti-human CD235a-PE	Biolegend	349106	1:100
anti-human CD41-PE-Cy5	Biolegend	303708	1:100
human CD31/PECAM-1 Mab (clone 9G11)	R&D	BBA7	1:200
DAPI	Thermo	62248	1:5000
Alexa Fluor 568 goat anti-mouse	Invitrogen	A11004	1:500
